# Tuberculosis—making predictions, especially about the future

**DOI:** 10.1016/S1473-3099(17)30492-9

**Published:** 2017-08-18

**Authors:** Anna M Mandalakas, Frank Cobelens

**Affiliations:** Department of Pediatrics, Baylor College of Medicine, Houston, TX, USA (AMM); and Department of Global Health and Amsterdam Institute for Global Health and Development, Academic Medical Centre, Amsterdam, Netherlands (FC)

The End TB Strategy aims to reduce the burden of tuberculosis worldwide until global prevalence reaches that already attained by many developed countries. The goal amounts to 90% reduction in the incidence of tuberculosis and 95% reduction in tuberculosis deaths.^1^ The strategy focuses on early detection and treatment and preventive therapy. In 2015, 4·3 million patients with tuberculosis are estimated to have gone undetected and untreated globally,^2^ which highlights the need for expanding case finding among those most at risk. Evidence shows consistently that preventive therapy effectively reduces individual risk of tuberculosis.^3–5^ Expansion of preventive treatment beyond children, however, is hampered by poor ability to predict who will develop tuberculosis after exposure. Even among people with positive tuberculin skin test or interferon-γ-release assay results, no more than 2·7% will develop tuberculosis within 2 years. Thus, at least 37 individuals will need to be treated to prevent one case, which, clearly, is too many.^6^ Improved prediction, therefore, is essential for reaching the End TB Strategy targets.

In *The Lancet Infectious Diseases,* Matthew J Saunders and colleagues^7^ present a derivation and external validation cohort study of a risk score that effectively identified adult tuberculosis contacts at risk of developing the disease in two Peruvian communities. The score is based on nine established and readily collectable clinical and demographic risk factors for the disease that need no testing. Capitalising on 10 years of observational data for prevalence of tuberculosis and repeated fitting of 200 bootstrap samples from the original cohort to their risk model, the score stratified individuals by risk of tuberculosis in the derivation cohort. The 10-year risks were low 2·8% (95% Cl 1·7–4·4), medium 6·2% (4·8–8·1), and high 21% (17–24). Assuming 75% effectiveness of preventive therapy, these data suggest that the number of contacts who would need to be treated to prevent one case of tuberculosis would be 48 in the low-risk group, 22 in the medium-risk group, and six in the high-risk group. In the validation cohort, information from patients with tuberculosis was used to calculate and stratify 2·5-year risks among contacts, which were 1·4% (95% Cl 0·70–2·8) for low risk, 3·9% (2·5–5·9) for medium risk, and 8·6% (5·9–12·6) for high risk. These findings indicate the potential of this risk score as a practical tool to use in the field.

Saunders and colleagues eloquently demonstrate the usefulness of well recognised risk factors to identify contacts at risk in a region with medium tuberculosis incidence and low HIV prevalence. They further show the feasibility of using their risk score to target screening, surveillance, and preventive treatment among adult contacts, complementing the evidence supporting simple approaches to stratify risk among child contacts.^8,9^ However, although the study potentially represents a major step forward, there are some points of caution to consider. Based on data from the derivation cohort only, the authors surmise that their score predicts increased risk of tuberculosis for at least 10 years. Since the validation cohort had only 2·5 years of follow-up, this conclusion might have been made post hoc. Moreover, with an annual tuberculosis case notification rate of 199 per 100 000 people in the derivation cohort study area, 20% of tuberculosis cases occurring in a 10-year period might be due to reinfection, which a contact risk score is unlikely to predict. The cumulative risk of up to 8·6% seen in the validation cohort might, therefore, be more realistic than 21% in the derivation cohort. Nonetheless, Saunders and colleagues′ findings still suggest an important advance over existing approaches for risk stratification among adult contacts.

The risk score captures a combination of factors related to transmission and disease progression, which reflects the complex relation between characteristics of the community, the host, and the infecting *Mycobacterium tuberculosis* strain. These relations are setting specific, and, therefore, the predictive power of the risk score will be also. This issue seems exemplified by the weaker prediction in the external than in the internal validation. Recognised risk factors for tuberculosis, such as drug resistance, HIV, and diabetes, were not included in the risk score, but might need to be considered in populations with high prevalence. The risk score focused on household transmission, but exposure outside households is also suggested to contribute substantially to transmission in some communities.^10^ Scores that include community transmission would, therefore, also be useful. Additionally, scores that include housing characteristics and socioeconomic status might yield varying results in different populations.^11^ Saunders and colleagues′ risk score needs to be validated in a variety of populations and then adaptated to local conditions based on the findings. Finally, evaluation of preventive strategies for tuberculosis that target adults will be crucial to show cost-effectiveness and equal benefit across socioeconomic groups.^12^ Elimination of tuberculosis worldwide will need a global commitment to narrow the extensive case-detection gap and expand preventive efforts. Evidence-based and risk-based approaches to deliver preventive therapy can help to pave the path towards this goal.
